# Characterization of Dermal Stem Cells of Diabetic Patients

**DOI:** 10.3390/cells8070729

**Published:** 2019-07-16

**Authors:** Letizia Ferroni, Chiara Gardin, Luca Dalla Paola, Gianluca Campo, Paolo Cimaglia, Gloria Bellin, Paolo Pinton, Barbara Zavan

**Affiliations:** 1 Maria Cecilia Hospital, GVM Care & Research, 48033 Cotignola (RA), Italy; 2 Department of Medical Sciences, University of Ferrara, via Fossato di Mortara 70, 44121 Ferrara, Italy; 3 Department of Morphology, Surgery and Experimental Medicine, Section of Pathology, Oncology and Experimental Biology and Laboratory for Technologies of Advanced Therapies (LTTA), University of Ferrara, 44121 Ferrara, Italy

**Keywords:** diabetic foot ulcers, dermal stem cells, gene expression array, *ALDH1A1*

## Abstract

Diabetic foot ulcers (DFUs) are lesions that involve loss of epithelium and dermis, sometimes involving deep structures, compartments, and bones. The aim of this work is to investigate the innate regenerative properties of dermal tissue around ulcers by the identification and analysis of resident dermal stem cells (DSCs). Dermal samples were taken at the edge of DFUs, and genes related to the wound healing process were analyzed by the real-time PCR array. The DSCs were isolated and analyzed by immunofluorescence, flow cytometry, and real-time PCR array to define their stemness properties. The gene expression profile of dermal tissue showed a dysregulation in growth factors, metalloproteinases, collagens, and integrins involved in the wound healing process. In the basal condition, diabetic DSCs adhered on the culture plate with spindle-shaped fibroblast-like morphology. They were positive to the mesenchymal stem cells markers CD44, CD73, CD90, and CD105, but negative for the hematopoietic markers CD14, CD34, CD45, and HLA-DR. In diabetic DSCs, the transcription of genes related to self-renewal and cell division were equivalent to that in normal DSCs. However, the expression of *CCNA2*, *CCND2*, *CDK1*, *ALDH1A1*, and *ABCG2* was downregulated compared with that of normal DSCs. These genes are also related to cell cycle progression and stem cell maintenance. Further investigation will improve the understanding of the molecular mechanisms by which these genes together govern cell proliferation, revealing new strategies useful for future treatment of DFUs.

## 1. Introduction

Diabetes mellitus is a universal health problem. People suffering from diabetes endure long-term complications such as cardiovascular diseases, nephropathy, retinopathy, neuropathy, and ulcers in the lower limbs. Indeed, diabetic neuropathy and peripheral arterial disease are common factors that cause diabetic foot ulcers (DFUs). In particular, motor neuropathy causes muscle weakness, atrophy, and paresis, sensory neuropathy leads to loss of the protective sensation of pain, pressure, and heat, and autonomic dysfunction causes vasodilation and decreased sweating, resulting in a loss of skin integrity, providing a site vulnerable to microbial infection [1]. It is estimated that between 15% and 25% of patients suffering from diabetes mellitus develop during their lifetime skin ulcers below the ankle, which seriously affect their quality of life, can be limb- and life-threatening, and are responsible for the majority of hospital admissions among diabetics. DFUs constitute a major public health burden in both the developed and developing countries [2]. Current treatment of DFUs encompasses glycaemia control, appropriate wound dressing management, offloading, revascularization if critical limb ischemia is assessed, and when required, prompt surgical debridement. DFUs predispose patients to local infection, osteomyelitis, gangrene, systemic sepsis, and amputation [3]. The 5 year mortality rate associated with DFUs requiring amputation ranges from 39 to 80%, extremely close to the most aggressive forms of cancer [4]. Therefore, the research continuously investigates novel strategies to promote healing in the diabetic foot and reduce the morbidity and mortality.

DFUs can be defined as lesions that involve loss of epithelium and dermis, sometimes involving deep structures and bones. Several studies have focused on the investigation of granulation tissue in diabetic ulcers. A series of molecular alterations were observed in the healing process, including high concentration of metalloproteinases, nonphysiological inflammatory response, oxidative stress, deficient neoangiogenesis, insufficient concentrations of growth factors, and high probability of infection [5,6,7,8].

Adult mammalian dermis contains tissue-derived stem cells with a high proliferation potential and an expression profile similar to adult mesenchymal stem cells isolated from many other adult tissues [9]. The dermal stem cells (DSCs) are responsible for the high regenerative potential of the skin, and may represent a means for the purposes of regenerative medicine. The clinical utility of stem cells in caring for injuries is primarily based on repairing and replacing cellular substrates, attenuation of inflammation, increasing angiogenesis, and enhancing migration of reparative cells. Stem cells are sought after due to their unique ability to initiate different wound healing programs, depending on the environmental milieu [10,11,12].

The aim of this work is to investigate the innate regenerative properties of dermal tissue around ulcers by the identification and analysis of resident DSCs. Dermal samples were taken at the edge of DFUs, and the expression profile of genes associated to the wound healing process was analyzed by the real-time PCR array technique. In an effort to characterize resident stem cells, we isolated DSCs from the same samples and analyzed their morphology by immunofluorescence as well as flow cytometry. Furthermore, a real-time PCR array of stem cell markers was carried out to define stemness properties of diabetic DSCs.

## 2. Materials and Methods

### 2.1. Patients Recruitment and Samples Collection

The study was conducted at Maria Cecilia Hospital (Cotignola, Ravenna, Italy) following the ethical principles for medical research involving human subjects of the World Medical Association Declaration of Helsinki. The patients participated in the study after signing the consent form. Patients met the following inclusion criteria: older than age 18 years; known diagnosis of diabetes mellitus; assessment of critical limb ischemia and infection was carried out; signature of the consent form. The exclusion criteria were known or suspected cancer diagnosis, chronic renal failure in dialytic treatment, and life expectancy of less than 1 year.

The study involved 20 diabetic patients with critical ischemia of the lower limbs and ulcer. The management of the infected ulcer involved antibiotic therapy targeted to microbiological isolations and surgical debridement to remove the necrotic and infected area. During surgery, the discarded tissue was stored and destined for the laboratory investigations. The dermis at the edge of the ulcer was used for gene expression analysis by real-time PCR array and for the isolation and characterization of resident stem cells.

### 2.2. RNA Extraction and Real-Time PCR Array

Total RNA was extracted from dermis or cultured cells with the RNeasy Mini Kit (Qiagen, Hilden, Germany), including an on-column DNase digestion step by the RNase-Free DNase Set (Qiagen), according to the manufacture procedure. The concentration and purity of the RNA were checked by spectrophotometric measurement on the NanoDrop 2000 Spectrophotometer (Thermo Fisher Scientific, Waltham, MA, USA). Of each sample, 500 ng of total RNA was reverse-transcribed with RT^2^ First Strand Kit (Qiagen) in a SimpliAmp Thermal Cycler (Applied Biosystems, Thermo Fisher Scientific) following the manufacture procedures. The resultant first-strand cDNAs were stored at −20 °C until the next step. Human wound healing RT^2^ Profiler PCR Array and human stem cell RT^2^ Profiler PCR Array (Qiagen) were performed, in accord with the manufacture protocol. Briefly, the cDNA samples were mixed with RT^2^ SYBR Green Mastermix (Qiagen), and then aliquoted into the wells of the RT^2^ Profiler PCR Array. A StepOnePlus Real-Time PCR System (Applied Biosystems, Foster City, CA, USA) was set up with the following thermal cycling conditions: denaturation at 95 °C for 10 min; followed by 40 cycles of denaturation at 95 °C for 15 s; annealing and elongation at 60 °C for 1 min. A dissociation curve for each well was performed by running the following program: 95 °C for 1 min; 65 °C for 2 min; 65 °C to 95 °C at 2 °C/min. Relative expression was determined using the 2ΔΔCT method. Ct values of target genes were normalized to the geometric mean Ct values of five housekeeping genes (*ACTB*, *B2M*, *GAPDH*, *HPRT1*, and *RPLP0*). Results were reported as fold regulation of target genes in the test group (pathological) compared with the control group (healthy). Fold regulation values greater than 2 indicate increased gene expression, fold regulation values less than −2 indicate decreased gene expression, and fold regulation values between −2 and 2 indicate indifferently expressed genes. Data analysis was performed by using the GeneGlobe Data Analysis Center (Qiagen). The data analysis software does not perform any statistical analysis beyond the calculation of *p* values using Student’s t-test based on 2ΔCT values for each gene of the test group compared with the those of control group. Statistical significance was set at *p* < 0.05.

### 2.3. Cells Isolation and Characterization

Dermis was removed from DFU biopsy and washed in phosphate-buffered saline (PBS, EuroClone, Milano, Italy) added with 1% antibiotic–antimycotic (AA, Thermo Fisher Scientific, Waltham, MA, USA). Dermal tissue was minced and digested with 200 U/mL collagenase type II (Gibco, Thermo Fisher Scientific) in Hanks’ balanced salts solution (HBSS, Euroclone) at 37 °C for 16 h. The resulting cells were pelleted, rinsed with PBS, and counted using the trypan blue exclusion assay. They were seeded at a density of 5 × 10^4^ cells/cm^2^ in Basal Medium (BM) consisting of Dulbecco’s modified Eagle’s medium (DMEM, Euroclone) supplemented with 10% Fetal Bovine Serum (FBS, EuroClone), and 1% AA. Cell cultures were maintained at 37 °C and 5% CO_2_, and medium was changed twice a week.

Cells within 3–5 passages were harvested by trypsin treatment (trypsin/EDTA, EuroClone), then counted under Bürker Chamber (Paul Marienfeld GmbH & Co. KG, Lauda-Königshofen, Germany).

For immunofluorescence staining, 2 × 10^4^ cells/cm^2^ were seeded on glass coverslips put into 24 well plates and cultured in BM. The following day, cells were fixed in 4% paraformaldehyde (Sigma-Aldrich, St. Louis, MO, USA) for 10 min. After three washes, cells were incubated in 3% bovine serum albumin (BSA; Sigma-Aldrich) solution in PBS at room temperature (RT) for 1 h. Then, cells were incubated overnight at 4 °C with the primary antibodies: mouse anti-human CD44 (Thermo Fisher Scientific), rabbit anti-human CD73 (Abcam, Cambridge, UK), rabbit anti-human CD90 (Abcam, Cambridge, UK), and mouse anti-human CD105 (Thermo Fisher Scientific). Then, cells were incubated with the fluorescent secondary antibodies goat anti-rabbit Alexa Fluor 555 (Thermo Fisher Scientific), or goat anti-mouse Alexa Fluor 488 (Thermo Fisher Scientific) at RT for 1 h. Actin staining was performed with Phalloidin Alexa Fluor 555 (Thermo Fisher Scientific) in PBS for 20 min at RT, and nuclear staining with NucBlue Fixed Cell Stain (4′,6-diamidin-2-fenilindolo, DAPI; Thermo Fisher Scientific) in PBS for 5 min at RT. Immunofluorescence images were acquire on an Upright ECLIPSE Ni Microscope (Nikon, Minato, Tokyo, Japan).

For flow cytometry, as previously described [13], cells were dissociated and resuspended in flow cytometry staining buffer (R&D Systems, Minneapolis, MN, USA) at a final cell concentration of 1 × 10^6^ cells/mL. Cells were incubated with the following fluorescent monoclonal mouse anti-human antibodies (eBioscienceTM, Thermo Fisher Scientific): CD14 R-PE; CD34 FITC; CD44 FITC; CD45 APC; CD73 APC; CD90 R-PE; CD105 PE-Cy 7; HLA-DR FITC. Cells were washed twice with 2 mL of flow cytometry staining buffer and resuspended in 500 µL of flow cytometry staining buffer. Flow cytometry analyses were performed on an Attune NxT flow cytometer (Thermo Fisher Scientific) with the Attune NxT software (Thermo Fisher Scientific). Each experiment was performed independently three times. Results were expressed as mean ± standard deviation (SD).

Cell growth has been investigated by the cumulative population doubling (CPD) assay. Briefly, 1.2 × 10^5^ cells at passage 2 (p2) were seeded into 6 well plates. Every two days, cells were detached, counted, and seeded again at the same density in a new 6 well plate. This was repeated until the cells reached p6. The population doubling (PD) of the cells was calculated according to the formula:

PD = (logN_t_ − logN_0_)/log2 (1)

where PD represents the number of cell divisions that occur in each passage; N_t_ corresponds to cell number on the second day, and N_0_ is the initial seeding number of cells. To determine the CPD, the PD level for each passage was calculated and added to the levels of the previous passages. The experiment was performed independently three times. Results were expressed as mean ± standard deviation (SD). Student’s t-test was performed to determine the statistical significance. Statistical significance was set at *p* < 0.05.

Cell migration ability was investigated by in vitro wound healing assay. Briefly, 2 × 10^4^ cells were seeded in 24 well plates and cultured until they reached confluence. Cell monolayers were scratched by a 100 µL sterile pipette tip. After a wash with PBS, fresh BM was added. Images of the whole wound were taken immediately after the scratch and after 24 h with a Nikon Inverted Microscope Eclipse Ti-E equipped with a Digital Sight camera DS-03. Scratch areas were measured at 0 and 24 h, and migration rate was calculated according to the formula:

Migration (%) = 100 × (A_0_ − A_t_)/A_0_ (2)

where A**_0_** corresponds to whole wound taken immediately after the scratch, and A**_t_** corresponds to whole wound taken after 24 h. At least three independent experiments were performed. Results were expressed as mean ± standard deviation (SD). Student’s t-test was performed to determine the statistical significance. Statistical significance was set at *p* < 0.05.

## 3. Results

### 3.1. Gene Expression Profile of Diabetic Dermis

**Figure 1:**
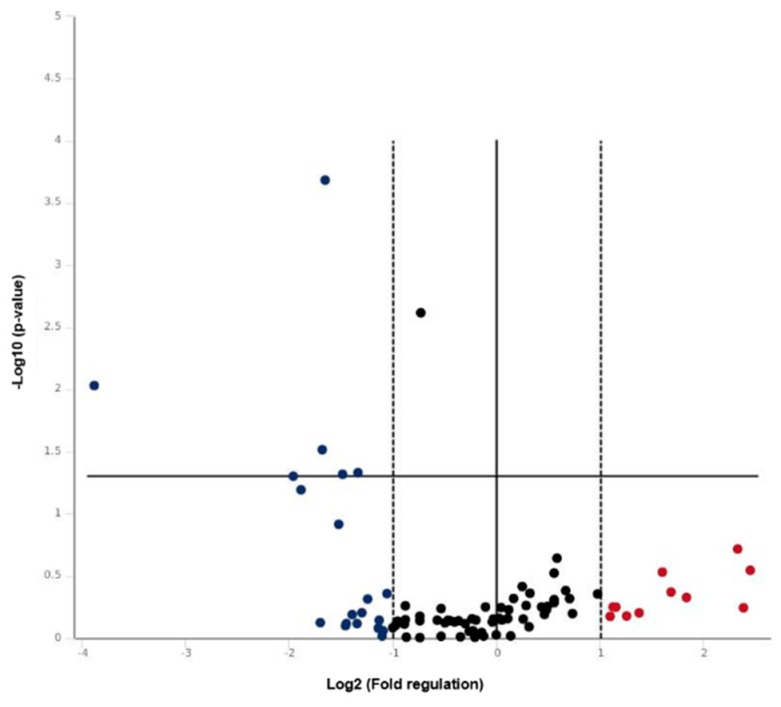
Volcano plot of the wound healing real-time PCR array results. The vertical black line indicates a 1.0-fold change in gene expression. The vertical dashed lines indicate the desired threshold of a 2.0-fold change in gene expression. The horizontal black line indicates the desired 0.05 threshold for the *p*-value of the t-test.

In order to define the molecular alterations underlying the development of chronic ulcers in diabetic patients, the expression of 84 key wound healing associated genes was probed by real-time PCR array. The array comprises genes expressed during the four phases of the inflammatory response: coagulation, inflammatory, proliferative, and maturation phase. The expression profile of wound healing genes of diabetic dermis taken from the edge of 20 ulcers was compared with that of normal dermis. Plotting of the 84 detected transcripts on a volcano plot (Figure 1) indicated that 31 genes were differentially expressed in diabetic dermis and healthy dermis by 2-fold or greater. A total amount of 11 genes was upregulated, whereas 20 genes were downregulated. The complete list of genes investigated and the fold regulation values are reported in Table 1.

Among the genes involved in the coagulation phase, coagulation factor III (*F3*), fibrinogen alpha chain (*FGA*), and plasminogen (*PLG*) were downregulated, whereas plasminogen activator urokinase receptor (*PLAUR*) was upregulated. Transcripts of inflammatory chemokines and cytokines, such as chemokine (C-C motif) ligand 2 (*CCL2*), chemokine (C-X-C motif) ligand 1 (*CXCL1*), chemokine (C-X-C motif) ligand 2 (*CXCL2*), and interleukin 1 beta (*IL1B*) were upregulated, and colony stimulating factor 2 (*CSF2*) and interleukin 2 (*IL2*) were downregulated. The proteolytic enzymes cathepsin G (*CTSG*), matrix metallopeptidase 1 *(MMP1*), and matrix metallopeptidase 9 (*MMP9*) were upregulated, whereas matrix metallopeptidase 2 (*MMP2*) downregulated. Overall, the proteins constituting the extracellular matrix showed a reduction in the gene expression profile. In particular, collagen type IV alpha 3 (*COL4A3*), collagen type V alpha 3 (*COL5A3*), and vitronectin (*VNT*) were downregulated. Also cell adhesion molecules integrin alpha 1 (*ITGA1*), integrin alpha 2 (*ITGA2*), integrin beta 3 (*ITGB3*) showed a downregulation. Moreover, the expression of growth factors registered a broad downregulation: fibroblast growth factor 2 (*FGF2*), platelet-derived growth factor (*PDGF*), connective tissue growth factor (*CTGF*), and colony stimulating factor 2 (*CSF2*) were particularly downregulated.

### 3.2. Stem Cell Isolation and Morphological Characterization

**Figure 2:**
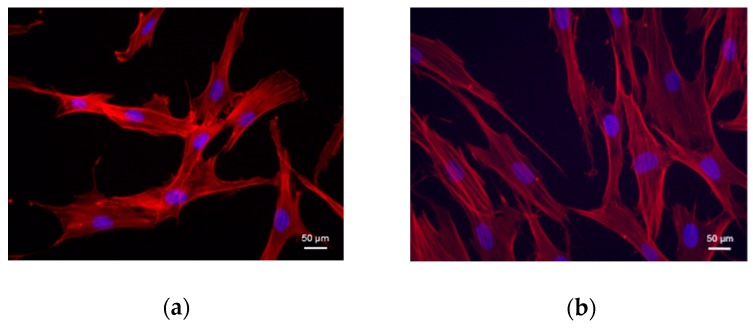
Morphology of cells isolated from (**a**) diabetic dermis and (**b**) normal dermis. The immunostaining of actin filaments with phalloidin (red) shows a spindle-shaped fibroblast-like morphology. Cell nuclei are counterstained in blue with DAPI (magnification 40×).

Cells were isolated from diabetic and normal dermis by enzymatic digestion and plated under basal conditions in cell culture flasks. Like the normal cells, the diabetic cells have adhered to flask’s plastic forming a monolayer. The immunostaining of actin filaments with phalloidin revealed a spindle-shaped fibroblast-like morphology both in diabetic and in normal cells (Figure 2).

**Figure 3:**
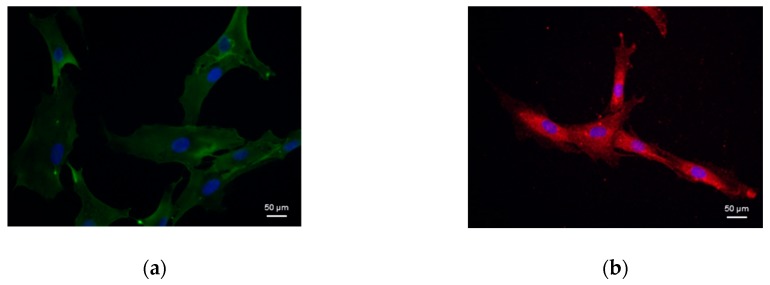

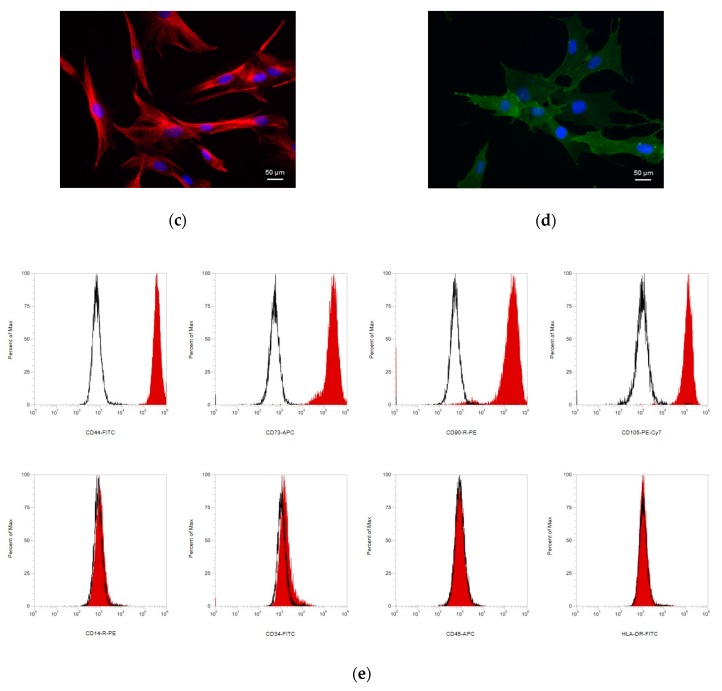
Characterization of cells isolated from diabetic dermis. Immunofluorescent staining of: (**a**) CD44 (in green), (**b**) CD73 (in red), (**c**) CD90 (in red), (**d**) CD105 (in green). Nuclei are stained with DAPI in blue (magnification 40×). (**e**) Detection of cell surface markers by flow cytometry: cells are positive to CD44, CD73, CD90, and CD105, and negative to CD14, CD34, CD45, and HLA-DR.

The cell surface antigens were characterized by immunofluorescence and flow cytometry analyses (Figure 3). Diabetic cells were positive to immunofluorescent staining with CD44 (in green, Figure 3a), CD73 (in red, Figure 3b), CD90 (in red, Figure 3c), and CD105 (in green, Figure 3d). Flow cytometry confirmed that diabetic cells were positive to the mesenchymal stem cells markers CD44, CD73, CD90, and CD105, but negative for the hematopoietic markers CD14, CD34, CD45, and HLA-DR (Figure 3e). The percentages of isolated cells expressing the cell surface markers are reported in Table 2.

**Figure 4:**
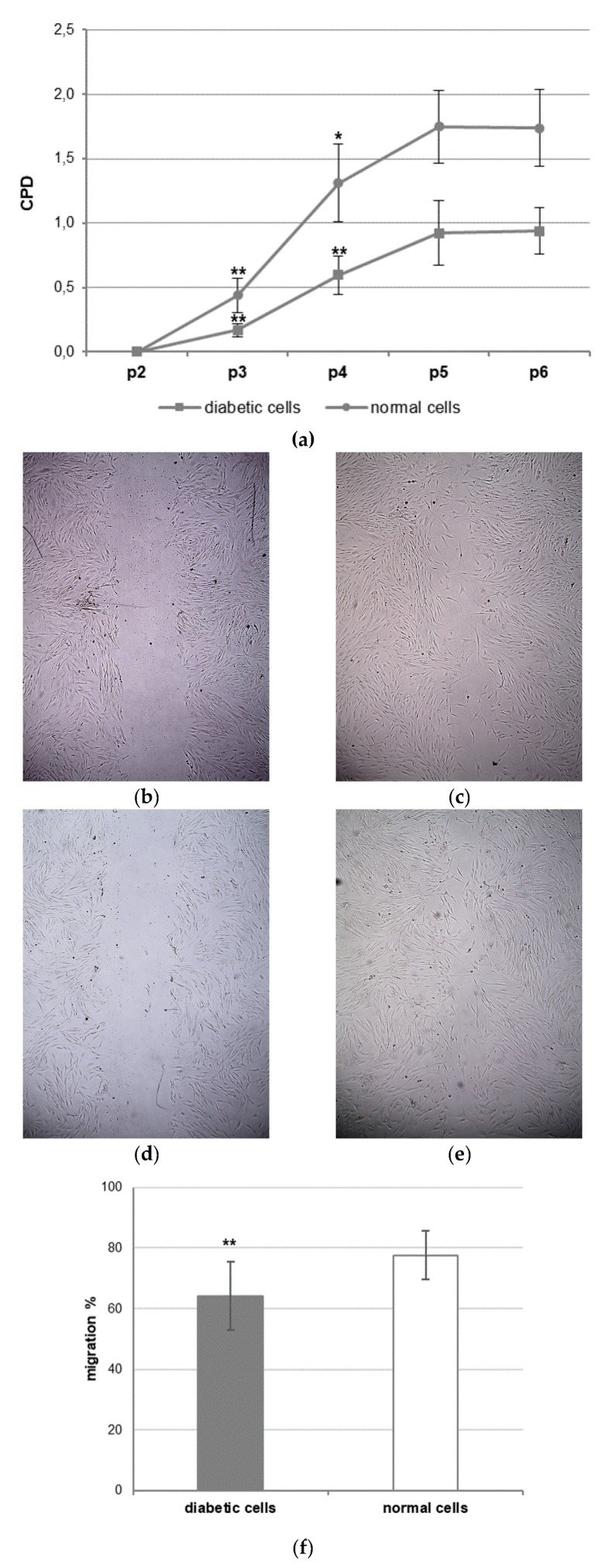
Growth and migration of diabetic dermal cells compared with those of normal dermal cells. (**a**) Cumulative population doubling (CPD) of diabetic cells (square indicator) and normal cells (round indicator). (**b**–**e**) In vitro wound healing assay: representative images (10x magnification) of the whole wound area taken in the scratch assay for (**b**) diabetic cells at 0, (**c**) diabetic cells at 24 h, (**d**) normal cells at 0, (**e**) normal cells at 24 h. (**f**) The migration percentages (%) are expressed as mean ± standard deviation (SD). * *p* < 0.05, ** *p* < 0.01.

The rate of growth and migration of diabetic cells was compared with that of normal cells (Figure 4). A CPD assay was performed to establish growth potential of diabetic and normal dermal cells during five consecutive passaging (from p2 to p6). The CPD corresponds to the total number of estimated divisions during the considered interval. The CPD tended to be lower for diabetic cells with respect to normal cells at all passages examined (Figure 4a). The migration rate was investigated by in vitro wound healing assay (Figure 4b–f). Even the migration rate of diabetic cells was lower than that of normal cells (Figure 4f).

### 3.3. Gene Expression Profile of Diabetic Dermal Cells

**Figure 5:**
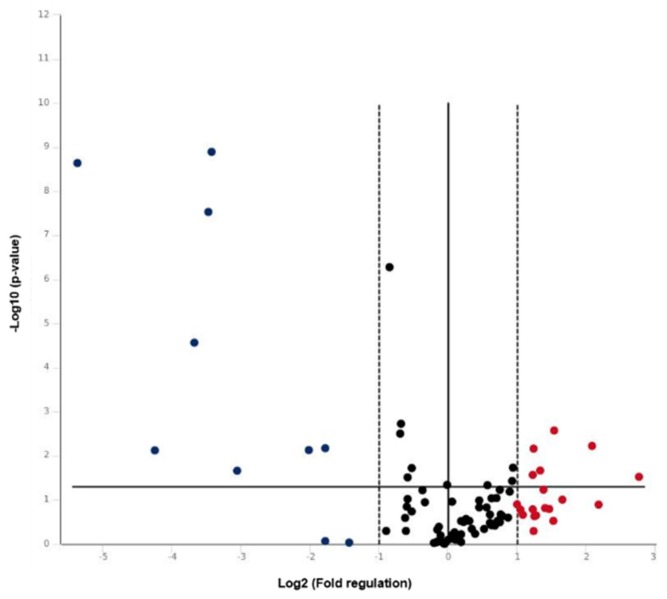
Volcano plot of the stem cell real-time PCR array results. The vertical black line indicates a 1.0-fold change in gene expression. The vertical dashed lines indicate the desired threshold of a 2.0-fold change in gene expression. The horizontal black line indicates the desired 0.05 threshold for the *p*-value of the t-test.

The expression profile of genes characterizing stem cells was investigated in diabetic dermal cells by real-time PCR array. The array comprises genes that control self-renewal, cell division, cell cycle, cell communication, cell adhesion, metabolism, and differentiation. The transcripts of diabetic cells were compared with those of healthy DSCs. The volcano plot in Figure 5 shows that 29 genes were differentially expressed in diabetic DSCs and healthy DSCs by 2-fold or greater. In particular, 19 genes were upregulated and 10 were downregulated. The fold regulations of the 84 investigated genes are reported in Table 3.

In diabetic DSCs, the genes related to self-renewal, symmetric, and asymmetric cell division showed a comparable expression profile to those of healthy DSCs. The transcription of *NOTCH1*, *NOTCH2*, *WNT1*, *SOX2*, and desert hedgehog (*DHH*) were equivalent to that in normal DSCs. In addition, the genes involved in the WNT signaling including *APC, AXIN1*, cyclin D1 (*CCND1*), frizzled family receptor 1 (*FZD1*), *FRAT1*, and *MYC* were equally expressed. The genes connected to the Notch signal were investigated: the transcription of delta-like 1 (*DDL1*) gene was equivalent to that in normal DSCs, whereas jagged1 (*JAG1*) and deltex 2 (*DXT2*) transcription was upregulated. The transcription of genes regulating cell cycle progression, such as cyclin A2 (*CCNA2*), cyclin D2 (*CCND2*), and cyclin-dependent kinase 1 (*CDK1*) were downregulated. The gene expression of growth factors, including fibroblast growth factor 1 (*FGF1*), fibroblast growth factor 2 (*FGF2*), and fibroblast growth factor 3 (*FGF3*), was equal in diabetic DSCs compared to normal DSCs. Instead the expression of bone morphogenetic protein 1 (*BMP1*), bone morphogenetic protein 3 (*BMP3*), and growth differentiation factor 2 (*GDF2*, alias *BMP9*) was upregulated in diabetic DSCs. Moreover, the gene expression of the metabolism genes such as ATP-binding cassette sub-family G member 2 (*ABCG2*) and aldehyde dehydrogenase 1 family member A1 (*ALDH1A1*) were downregulated.

## 4. Discussion

Skin wound healing is a dynamic process tightly regulated. The regular order in which tissue healing takes place is divided into four stages, corresponding roughly to the four waves of predominant cell type appearing in the wound bed. These are the hemostasis, inflammatory, proliferative, and maturation phases [14]. In the hemostasis phase, platelets and circulating coagulant factors accumulate at the site of tissue injury. The blood clotting is achieved by a cascade of enzymatic reactions, which involves prothrombin and a series of factors, which are converted to active proteases by hydrolysis. F3 is the primary initiator of the extrinsic blood coagulation. Upon injury of the vessel and surrounding tissue, F3 is exposed to blood coagulation factors. The complex with coagulation factor VII catalyzes the proteolytic activation of the coagulation factors X and IX, leading to thrombin generation [15]. Thrombin converts the soluble protein FGA to an insoluble fibrin gel. Thrombin also activates Factor XIII (F13A1), which crosslinks the fibrin polymers and forms a fibrin mesh that traps circulating platelets, leucocytes, and red blood cells [16]. The actions of thrombin and several other activated coagulation factors are inhibited by circulating antithrombin, e.g., serpin peptidase inhibitor clade E member 1 (SERPINE1). Once sufficient thrombin is produced to overcome the effect of circulating antithrombin, the coagulation is able to initiate. The clot dissolution is carried out by plasmin, which catalyzes the fibrin hydrolysis. Plasmin is generated from PLG by extracellular protease plasminogen activator urokinase (PLAU) and tissue-type PLAU (PLAT) that are directly activated and released in extracellular matrix (ECM) by a number of growth factors, e.g. hepatocyte growth factor (HGF) [17]. PLAU also plays a pivotal role in cell adhesion and migration by binding *PLAUR*. Even a modest increase in *PLAUR* expression is correlated with the ability of monocytes to penetrate stromal tissue in a PLAU-dependent process. In addition, *PLAUR* mediates cellular adhesion to VTN, promotes integrin-dependent migration, and initiates intracellular signaling events [18]. In diabetic dermis, the enzymes involved in the creation (*F3* and *FGA*) and the dissolution (*PLG*) of fibrin clot were downregulated. On the contrary, *PLAUR* was upregulated to favor the migration of monocyte in the site of injury. The inflammatory phase is characterized by immune cells infiltration. Neutrophils are initially captured from the fast-flowing blood stream in the postcapillary microvasculature to the endothelial vessel wall, followed by rolling along the endothelium, integrin-dependent arrest and firm adhesion, intravascular crawling, and trans-endothelial migration into the inflamed tissue. Inflammatory stimuli, such as tumor necrosis factor alpha (TNFα) and lipopolysaccharide (LPS), induce the upregulation of adhesion molecules on the surface of platelets and vascular endothelial cells that enables these cells to stick to each other and to neutrophils [19]. The migration, adhesion, and activation of leucocytes also depends on the expression of specific integrins. Each integrin consists of one α (ITGA) subunit and one β (ITGB) subunit. Leucocytes by integrin α4β1 (alias CD49d/CD29) bind vascular cell adhesion protein 1 (VCAM-1) and FGA, whereas integrin αVβ3 (alias CD51/CD61) binds intercellular adhesion molecule 1 (ICAM-1), platelet endothelial cell adhesion molecule (PECAM-1), VCAM-1, VTN, and FGA [20]. Compared with the control condition, in the diabetic dermis the expression of *ITGB3* was downregulated while *ITGA4*, *ITGAV*, and *ITGB1* genes were indifferently expressed. This could be related to a lesser lymphocyte activation and a lesser adhesion of them to platelets and endothelial cells. In addition to physical contacts, platelets secrete proinflammatory chemokines such as, *CXCL1* with a chemotactic activity for neutrophils, chemokine (C-X-C motif) ligand 5 (*CXCL5*) a neutrophils activator, *CCL2* a potent chemoattractant for monocytes and basophils, and chemokine (C-C motif) ligand 7 (*CCL7*) a chemotactic factor for monocytes and eosinophils. Platelets also release soluble CD40 ligand (CD40L), which induces in endothelial cells the expression of inflammatory adhesion receptors (e.g., E-selectin, VCAM-1, ICAM-1), the production of chemokines and interleukins (ILs) (e.g., *CCL2*, *IL6*, and *IL8*), and the production of *MMP9* [21]. Moreover, immune cells, including monocytes, macrophages, and lymphocytes secrete many proinflammatory cytokines and growth factor to amplify the inflammatory response. Proinflammatory ILs (e.g., *IL1B*, *IL6*, and *IL8*) and chemokines (e.g., *CXCL2*, *CXCL11*), TNFα, *CSF2*, colony-stimulating factor 3 (*CSF3*) are released to stimulate cytokine production, cell proliferation, macrophage activation, and increase neutrophil and monocyte function [22,23]. Compared with in the control group, the dermis of diabetic patients showed an upregulation of the pro-inflammatory chemokines and cytokine (e.g. *CCL2*, *CXCL1*, *CXCL2*, and *IL1B*), but a downregulation of *CSF2* and *IL2*. *CSF2* can enhance macrophage proliferation as well as modulate their differentiation and function, while *IL2* modulates T-cell proliferation and activation. Despite an increase in chemokine expression, the downregulation of *CSF2* and *IL2* in diabetic dermis could underline an impairment in cell-mediated immune response. However, in diabetic dermis, the serine protease *CTSG* was upregulated. CTSG is released by activated neutrophils to clear pathogens and regulate inflammation by modifying chemokines, cytokines, and cell surface receptors. *CTSG* changes *CXCL5* and chemokine (C-C motif) ligand 15 (*CCL15*) into more potent chemotactic factors by a proteolytic processing. In addition, *CTSG* causes cell shape modification and intercellular gap formation in endothelial cell that increase endothelium permeability, supporting the migration of neutrophils, monocytes and antigen-presenting cells in the injured site [24]. *CTSG* affects also tissue remodelling directly by degrading components of the matrix and indirectly by cleaving and activating matrix metalloproteinases (MMPs), such as *MMP1* and *MMP2* [24]. Activated neutrophils also release many angiogenic molecules, such as *IL8*, *CXCL1*, and *MMP9*. In particular, *MMP9* and *IL8*, by degrading the basement membrane and promoting the endothelial cells migration, respectively, are responsible for the proangiogenic effects of neutrophils [25]. MMPs and their tissue inhibitors (TIMPs) are involved in various stages of the wound healing process, and their expression is regulated by several growth factors, including TNFα, transforming growth factor beta 1 (*TGFB1*), insulin-like growth factor 1 (*IGF1*), *PDGF*, epidermal growth factor (EGF), *FGF2*, vascular endothelial growth factor A (*VEGFA*), *IL6*, and *IL10* [26]. During the inflammatory phase, MMPs activate or inhibit several cytokines and improve leukocyte invasion, creating a chemotactic gradient. Several chemokines, including *CCL*7 and chemokine (C-X-C motif) ligand 12 (*CXCL12*) are substrates for *MMP2*. *MMP9* cleaves and activates chemokine (C-X-C motif) ligand 6 (*CXCL6*) and *IL8*, whereas it inactivates *CXCL1* and chemokine (C-X-C motif) ligand 4 (*CXCL4*) [27]. The gelatinase *MMP9* and *MMP2* are also responsible for the breaking down of type IV collagen in the basement membrane, thus are involved in keratinocyte migration and re-epithelialization [26]. In diabetic dermis, the expression of *MMP9* gene was upregulated, while their inhibitor *TIMP1* was indifferently expressed. Previous studies on DFUs have reported that a high concentration of *MMP9* in wound liquid and a high ratio of *MMP9* to TIMP1 are related to poor wound healing [28,29]. A 5-fold increase in the expression of *MMP1* was also detected in diabetic dermis. *MMP*1 is responsible for proteolytic degradation of type-1 and type-3 collagens as well as elastic fibres. It is crucial for wound re-epithelialization, and their dysregulation is associated to several pathological conditions, including cutaneous ulcer [30]. In diabetic dermis, the upregulation of *CTSG*, *MMP9*, and *MMP1* may depend on bacterial infection at the site of DFU and on a dysregulation of growth factors. Compared with the healthy control, diabetic dermis showed a downregulation of various growth factors, particularly *PDGF*, *FGF2*, and *CTGF*. *PDGF* works as a chemoattractant for inflammatory cells and fibroblasts. It induces fibroblast migration and proliferation, matrix production, production of granulation tissue proteins and provisional ECM, and angiogenesis during the healing process. Various clinical studies have shown that a downregulation of *PDGF* and its receptor are associated to an enhanced healing time [26]. *FGF2* stimulates the growth and differentiation of fibroblasts, vascular smooth muscle cells, and endothelial cells. It increases the rate and degree of granulation tissue formation, stimulating the healing process [31]. The formation of new blood vessels in new-formed tissue is initiated by *PDGF*, *FGF2*, as well as *VEGFA* [32]. Instead, the production of ECM and the inhibition of proteases are influenced predominantly by the fibrogenic growth factors, including *PDGF*, *IGF1*, and *TGFB1* [33]. *CTGF* modulates the interaction of cells with the ECM, promoting collagen deposition, mesenchymal cell activation and differentiation, and tissue remodelling [34]. The balance between matrix degradation and matrix formation is crucial in the wound healing process. In the diabetic dermis, different types of collagen were downregulated, including *COL4A3*, *COL5A3*, and *VTN*. Type IV collagen is the major structural component of basement membranes. Type V collagen regulates the assembly of heterotypic fibers composed of both type I and type V collagen. Therefore, the collagen fibers involved in the organization of the ECM, in angiogenesis and re-epithelialization appear to be compromised in diabetic dermis. VTN interacts with glycosaminoglycans and proteoglycans and is recognized by certain integrins and serves as a cell-to-substrate adhesion molecule. In diabetic dermis, even the *ITGA1*, *ITGA2*, and *ITGB1* integrin subunits able to bind collagens are downregulated. Smooth muscle cells, fibroblasts, and microvascular endothelium express integrin α1β1 to bind type IV collagen, whereas integrin α2β1 has predominantly epithelial distribution, showing a preference for type I collagen [35]. Among investigated integrins, only *ITGB6* gene showed upregulation in diabetic dermis. However, integrin αvβ6 is an epithelium-restricted molecule expressed at low levels in healthy skin that is rapidly upregulated in response to inflammation and injury [36].

Overall, the gene expression profile of proximal dermis to DFUs showed a dysregulation in growth factors, MMPs, collagens, and integrins involved in the proliferation phase, angiogenesis, re-epithelialization, and remodeling. In this scenario, the resident stem cells of dermal tissue around DFUs were isolated and analyzed. The cells of diabetic dermis comply with the parameters of dermis stem cells (DSCs) defined by Vapniarsky and colleagues [37], although they showed a lower proliferation and migration rates compared with the cells of nondiabetic DSCs. Diabetic DSCs have adhered to culture flasks with a spindle-shaped fibroblast-like morphology under the basal condition. Moreover, in basal medium diabetic DSCs were positive to the mesenchymal stem cells markers CD44, CD73, CD90, and CD105, but negative for the hematopoietic markers CD14, CD34, CD45, and HLA-DR.

Despite the morphology and phenotype of diabetic DSCs reflecting that of mesenchymal stem cells, the expression profile of stemness genes in diabetic DSCs showed some differences compared with that in DSCs of nondiabetic donors. Stem cells are defined as cells able to continuously divide to produce more undifferentiated stem cells, the so-called self-renewing property, and to differentiate into specialized cells, namely multilineage differentiation potency [38]. These properties are closely related to the ability of stem cells to proliferate by symmetric and asymmetric division. Asymmetric cell division gives rise to two daughter cells with distinct cell fates: one daughter maintains stem cell properties and functions, while the other daughter loses these characteristics. Symmetric cell division enables stem cells to generate two daughter cells having less potency than the parental stem cell. This mode of division is critical for expanding stem cell reservoirs, leading to rapid production of cells but potential depletion of the stem cell pool. Both symmetric and asymmetric cell divisions are employed in vivo to maintain the fine balance between self-renewal and differentiation of stem cells [39]. Notch, WNT, FGF, BMP, and DHH signaling play a key role in maintaining tissue homeostasis by regulating self-renewal of stem cells as well as proliferation or differentiation of progenitor cells [40]. In diabetic DSCs, the transcription of several genes related to cell division and self-renewal, including *NOTCH1*, *NOTCH2*, *WNT1*, *SOX2*, and *DHH* was equivalent to that in normal DSCs. The gene expression of growth factors, including *FGF1*, *FGF2*, and *FGF3*, involved in cell proliferation and differentiation was equal in diabetic DSCs as compared with normal DSCs. Instead, the expression of *BMP1*, *BMP3*, *BMP9* (alias *GDF2*) was upregulated in diabetic DSCs.

Notch1 and Notch2 coordinately maintain the stem cell pool in the quiescent state by preventing activation [41], whereas WNT proteins promote the proliferation of stem cells and regulate stem cell fate [42]. The WNT proteins, by promoting the ß-catenin translocation in nucleus, activate the transcription of target genes involving cell proliferation control, including *FGF2*0, DKK1, WISP1, MYC, and *CCND1* [43]. The expression of *MYC* and *CCND1* genes in diabetic DSCs was like to that in normal DSCs. Moreover, in diabetic DSCs the transcription of *FZD1* and *FRAT1* genes (positive regulators of the canonical pathway of Wnt signaling), and of *APC* and *AXIN1* genes (negative regulators) was equivalent to that in normal DSCs.

Asymmetric cell divisions can be controlled by Notch signaling through the activating ligands Jagged1 (*JAG1*) and Delta-like 1 (*DDL1*), and the negative regulator Deltex (*DXT2*) [40,44]. Compared to normal DSCs, the transcription of *DDL1* gene was equal in diabetic DSCs, whereas *JAG1* and *DXT2* transcription was upregulated.

Cell division and proliferation are also dependent by cyclins (CNNs) and cyclin-dependent protein kinases (*CDK*s), which together control cell cycle progression. In diabetic DSCs, the transcription of *CCNA2*, *CCND2*, and *CDK1* was downregulated compared with in normal DSCs. The cell cycle is a tightly regulated process that orchestrates genome duplication and accurate distribution of DNA and other factors into daughter cells after mitosis. *CCNA2* binds to and activates its catalytic partners, *CDK2* and *CDK1*. These complexes phosphorylate proteins like pocket proteins (RB, p107, p130) and proteins involved in DNA synthesis, thereby driving S-phase progression. In line with its role in regulating S phase, *CCNA2* expression is induced upon entry into S phase, persists through the S and G2 phases, and is degraded upon entry into mitosis. During early mitosis, *CCNA2* associates with *CDK1* and drives chromosome condensation and nuclear envelope breakdown [45]. Instead, *CCND2* regulating *CDK4* and *CDK6* drives the G1-to-S phase transition of the cell cycle [46]. Several works report that cyclins and *CDK*s are upregulated in different tumors such as breast, liver, and lung cancers, and the downregulation of them represents a good strategy to counteract the proliferation of the cancer stem cells [47,48]. Instead, the expression of cyclins and *CDK*s in stem cells had not yet been investigated.

The proliferation and differentiation of stem cells are also influenced by the activity of enzymes such us *ALDH1A*1 and *ABCG*2, which are downregulated in diabetic DSCs. Aldehyde dehydrogenase (ALDH) is considered a biomarker for stem cells, and its expression is also thought to closely correlate with the stemness of cancer stem cells [49]. ALDHs are family members of NAD-dependent enzymes that catalyze the oxidation of aldehydes to acids. They are localized in the cytoplasm, mitochondria, or nucleus and have been implicated in a wide variety of biological processes, including the detoxification of exogenously and endogenously generated aldehydes and the metabolism of vitamin A, alcohol, and reactive oxygen species. Of these, *ALDH1A1* mainly catalyzes the conversion of retinaldehyde to retinoic acid (RA) in vitamin A metabolism. RA enters the nucleus, binds to and activates the RA receptors (RARs) or the retinoid X receptors (RXRs), which are nuclear transcription factors that promote target gene expression. The genes downstream of RA are involved in many important biological processes, including cell differentiation, proliferation, and lipid metabolism [50]. RXR functions as an obligate heterodimeric partner for multiple nuclear receptors including the peroxisome proliferator-activated receptor-gamma (*PPARG*). The nuclear receptor *PPARG* is a master regulator of adipogenesis that controls the expression of multiple genes within complex transcriptional networks [51]. In the absence of *ALDH1A1*, both adipogenesis in vitro and diet-induced fat formation in vivo are markedly impaired. *ALDH1A1* deficiency increases retinaldehyde levels that inhibits RXR and *PPARG* activation, inhibiting *PPARG*-induced adipogenesis [52]. In diabetic DSCs, adipogenic differentiation is also prevented by the downregulation of the *PPARG* gene. Moreover, *ALDH1A1* controls the cell proliferation by regulating notch signaling. Notch genes have been shown to directly activate cyclins and *CDK2*, which control the cell cycle progression. Li et al. demonstrated that the deletion of *ALDH1A*1 inhibits cell cycle progression and cell proliferation by the suppression of the Notch/*CDK2*/Cyclin pathway [53]. Moreover, *ALDH1A*1 isozyme has been shown to play an important functional role in maintaining cancer stem cells. In cancer stem cells, the overexpression of *ALDH1A*1 is associated with enhanced invasion, colony formation, and chemoresistance. It was demonstrated that the stable downregulation of *ALDH1A*1 isozyme alone dramatically decreased their ability to form colonies and to proliferate [54]. Therefore, the downregulation of *ALDH1A1* observed in diabetic DSCs could be the cause of a reduced proliferative capacity due to a downregulation of cyclin and *CDK*s. In addition, the *ABCG2* gene was downregulated in diabetic DSCs. The *ABCG2* transporters belong to the ATP Binding Cassette (ABC) superfamily of transporters and use ATP hydrolysis to catalyze the transport of a wide range of substrates from the intracellular to the extracellular milieu. The overexpression of *ABCG2* has been shown to promote stemness, while the loss of *ABCG2* expression has been shown to promote lineage commitment in several adult stem cells. While the importance of *ABCG2* in stem cell maintenance has been shown, the precise mechanism by which *ABCG2* prevents stem cells from undergoing differentiation is unknown [55]. It seems that the high *ABCG2* activity increases the levels of transcripts involved in stemness such as *MEF*, *SOX2*, *OCT4*, *ID1*, and *HES1*, and that the activity of *ABCG2* is required for maintaining these stem markers and self-renewal property via Notch-independent manner [56]. Both enzymes have been broadly studied in cancer stem cells, however, data concerning their expression profile in diabetic stem cells are not previously reported in literature.

Overall, the resident stem cells of dermal tissue around DFUs showed morphology and phenotype equal to normal DSCs. However, diabetic DSCs showed reduced proliferative and migration capability. This could be related to the downregulation of the *ALDH1A1* enzyme that inhibits cell cycle progression and cell proliferation. Only further investigations could explain the mechanism by which the inflammatory and ischemic condition, typical of DFU, may cause a downregulation of this enzyme.

## 5. Conclusions

In conclusion, the present study has demonstrated that in diabetic dermal tissue around DFUs there is an impairment in the gene expression profile of growth factors, metalloproteinases, collagens, and integrins involved in the wound healing process. Moreover, we have isolated and characterized the resident DSCs. Although diabetic DSCs have the same morphology and phenotype as normal DSCs, the gene expression has revealed a downregulation of cyclins, *CDK1*, *ALD1H1A*, and *ABCG2*. These proteins are related to cell cycle progression, cell proliferation, and stem cell maintenance. Further investigation will improve the understanding of the molecular mechanisms by which these proteins together govern cell proliferation and will reveal new strategies aimed at enhancing the expression or activity of these proteins that will be useful for future treatment of DFUs.

## Figures and Tables

**Table 1:** Wound-healing-related genes probed by real-time PCR arrays.

Symbol	Gene	Fold Regulation	*p*-Value
*ACTA2*	Actin, alpha 2,	−3.89	0.050
*ACTC1*	Actin, alpha 1	1.19	0.702
*ANGPT1*	Angiopoietin 1	−1.23	0.767
*CCL2*	Chemokine (C-C motif) ligand 2	5.01	0.192
*CCL7*	Chemokine (C-C motif) ligand 7	−1.14	0.717
*CD40LG*	CD40 ligand	−1.38	0.721
*CDH1*	Cadherin 1, type 1, E-cadherin	1.47	0.519
*COL14A1*	Collagen, type XIV, alpha 1	−1.67	0.666
*COL1A1*	Collagen, type I, alpha 1	−1.99	0.825
*COL1A2*	Collagen, type I, alpha 2	−1.83	0.982
*COL3A1*	Collagen, type III, alpha 1	1.03	0.568
*COL4A1*	Collagen, type IV, alpha 1	−1.08	0.562
*COL4A3*	Collagen, type IV, alpha 3	−2.54	0.765
*COL5A1*	Collagen, type V, alpha 1	−1.97	0.786
*COL5A2*	Collagen, type V, alpha 2	1.48	0.491
*COL5A3*	Collagen, type V, alpha 3	−2.14	0.962
*CSF2*	Colony stimulating factor 2	−14.71	0.009
*CSF3*	Colony stimulating factor 3	−1.29	0.727
*CTGF*	Connective tissue growth factor	−3.22	0.031
*CTNNB1*	Catenin	1.40	0.557
*CTSG*	Cathepsin G	3.22	0.427
*CTSK*	Cathepsin K	−1.33	0.741
*CTSV*	Cathepsin L2	1.10	0.959
*CXCL1*	Chemokine (C-X-C motif) ligand 1	5.23	0.570
*CXCL11*	Chemokine (C-X-C motif) ligand 11	1.09	0.590
*CXCL2*	Chemokine (C-X-C motif) ligand 2	2.60	0.626
*CXCL5*	Chemokine (C-X-C motif) ligand 5	−1.86	0.771
*EGF*	Epidermal growth factor	−1.03	0.702
*EGFR*	Epidermal growth factor receptor	−3.15	0.000
*F13A1*	Coagulation factor XIII, A1 polypeptide	−1.67	0.724
*F3*	Coagulation factor III (thromboplastin)	−2.37	0.485
*FGA*	Fibrinogen alpha chain	−3.25	0.751
*FGF10*	Fibroblast growth factor 10	−1.37	0.723
*FGF2*	Fibroblast growth factor 2	−2.87	0.121
*FGF7*	Fibroblast growth factor 7	−1.16	0.984
*HBEGF*	Heparin-binding EGF-like growth factor	1.97	0.442
*HGF*	Hepatocyte growth factor	−1.94	0.731
*IFNG*	Interferon, gamma	1.08	0.696
*IGF1*	Insulin-like growth factor 1	3.03	0.294
*IL10*	Interleukin 10	1.03	0.704
*IL1B*	Interleukin 1, beta	3.57	0.472
*IL2*	Interleukin 2	−2.73	0.765
*IL4*	Interleukin 4	−1.48	0.718
*IL6*	Interleukin 6	1.59	0.415
*IL6ST*	Interleukin 6 signal transducer	−2.53	0.047
*ITGA1*	Integrin, alpha 1	−2.46	0.625
*ITGA2*	Integrin, alpha 2 (CD49B)	−2.08	0.439
*ITGA3*	Integrin, alpha 3 (CD49C)	−1.11	0.902
*ITGA4*	Integrin, alpha 4 (CD49D)	1.38	0.644
*ITGA5*	Integrin, alpha 5	−1.66	0.990
*ITGA6*	Integrin, alpha 6	−1.03	0.740
*ITGAV*	Integrin, alpha V (CD51)	−1.09	0.962
*ITGB1*	Integrin, beta 1 (CD29)	−1.17	0.873
*ITGB3*	Integrin, beta 3 (CD61)	−2.14	0.872
*ITGB5*	Integrin, beta 5	−1.41	0.755
*ITGB6*	Integrin, beta 6	2.13	0.668
*MAPK1*	Mitogen-activated protein kinase 1	−1.66	0.002
*MAPK3*	Mitogen-activated protein kinase 3	−1.45	0.967
*MIF*	Macrophage migration inhibitory factor	1.00	0.943
*MMP1*	Matrix metallopeptidase 1	5.45	0.285
*MMP2*	Matrix metallopeptidase 2	−2.62	0.648
*MMP7*	Matrix metallopeptidase 7	−1.84	0.711
*MMP9*	Matrix metallopeptidase 9	2.19	0.562
*PDGF*	Platelet-derived growth factor	−2.21	0.833
*PLAT*	Plasminogen activator, tissue	−1.36	0.724
*PLAU*	Plasminogen activator, urokinase	1.63	0.482
*PLAUR*	Plasminogen activator, urokinase receptor	2.23	0.563
*PLG*	Plasminogen	−2.20	0.719
*PTEN*	Phosphatase and tensin homolog	1.47	0.300
*PTGS2*	Prostaglandin-endoperoxide synthase 2	−1.18	0.699
*RAC1*	Ras-related C3 botulinum toxin substrate 1	1.12	0.480
*RHOA*	Ras homolog gene family, member A	1.02	0.693
*SERPINE1*	Serpin peptidase inhibitor, clade E, member 1	1.39	0.592
*STAT3*	Signal transducer and activator of transcription 3	1.19	0.385
*TAGLN*	Transgelin	−3.70	0.064
*TGFA*	Transforming growth factor, alpha	1.24	0.812
*TGFB1*	Transforming growth factor, beta 1	−1.84	0.551
*TGFBR3*	Transforming growth factor, beta receptor III	−2.80	0.048
*TIMP1*	TIMP metallopeptidase inhibitor 1	1.04	0.715
*TNF*	Tumor necrosis factor	1.22	0.548
*VEGFA*	Vascular endothelial growth factor A	1.35	0.562
*VTN*	Vitronectin	−2.75	0.794
*WISP1*	WNT1 inducible signaling pathway protein 1	1.66	0.635
*WNT5A*	Wingless-type MMTV integration site family, member 5A	2.39	0.664

*p*-values show the comparison results between the diabetic dermis and the healthy controls.

**Table 2:** Cell surface marker expression of cells isolated from diabetic dermis.

Surface Marker	% Expression
CD44	99,765 ± 0.340
CD73	99,805 ± 0.316
CD90	99,765 ± 1.613
CD105	98,468 ± 1.992
CD14	0.231 ± 0.248
CD34	0.098 ± 0.093
CD45	0.084 ± 0.092
HLA-DR	0.059 ± 0.041

Data are displayed as percentages expressed as mean ± standard deviation (SD).

**Table 3:** Stem cell-related genes probed by real-time PCR arrays.

Symbol	Gene	Fold Regulation	*p*-Value
*ABCG2*	ATP-binding cassette, sub-family G, member 2	−4.04	0.007
*ACAN*	Aggrecan	2.89	0.299
*ACTC1*	Actin, alpha, cardiac muscle 1	2.35	0.164
*ADAR*	Adenosine deaminase, RNA-specific	1.15	0.302
*ALDH1A1*	Aldehyde dehydrogenase 1 family, member A1	−41.08	0.000
*ALDH2*	Aldehyde dehydrogenase 2 family	1.86	0.065
*ALPI*	Alkaline phosphatase, intestinal	1.28	0.456
*APC*	Adenomatous polyposis coli	−1.50	0.031
*ASCL2*	Achaete-scute complex homolog 2	−1.85	0.505
*AXIN1*	Axin 1	1.63	0.090
*BGLAP*	Bone gamma-carboxyglutamate (gla) protein	1.48	0.148
*BMP1*	Bone morphogenetic protein 1	2.38	0.007
*BMP2*	Bone morphogenetic protein 2	−1.29	0.060
*BMP3*	Bone morphogenetic protein 3	2.07	0.165
*BTRC*	Beta-transducin repeat containing	1.20	0.271
*CCNA2*	Cyclin A2	−11.08	0.000
*CCND1*	Cyclin D1	1.14	0.604
*CCND2*	Cyclin D2	−2.70	0.930
*CCNE1*	Cyclin E1	−1.62	0.003
*CD3D*	CD3d molecule	−1.11	0.886
*CD4*	CD4 molecule	2.36	0.508
*CD44*	CD44 molecule	1.55	0.092
*CD8A*	CD8a molecule	1.69	0.320
*CD8B*	CD8b molecule	1.07	0.769
*CDC42*	Cell division cycle 42	−1.44	0.019
*CDH1*	Cadherin 1, type 1, E-cadherin	2.77	0.161
*CDH2*	Cadherin 2, type 1, N-cadherin	−1.11	0.903
*CDK1*	Cyclin-dependent kinase 1	−10.70	0.000
*COL1A1*	Collagen, type I, alpha 1	4.24	0.006
*COL2A1*	Collagen, type II, alpha 1	1.24	0.300
*COL9A1*	Collagen, type IX, alpha 1	1.52	0.215
*CTNNA1*	Catenin (cadherin-associated protein), alpha 1	1.00	0.808
*CXCL12*	Chemokine (C-X-C motif) ligand 12	−1.07	0.641
*DHH*	Desert hedgehog	1.44	0.456
*DLL1*	Delta-like 1	−1.52	0.506
*DLL3*	Delta-like 3	1.73	0.233
*DTX1*	Deltex homolog 1	1.14	0.903
*DTX2*	Deltex homolog 2	2.52	0.022
*DVL1*	Dishevelled, dsh homolog 1	1.68	0.059
*EP300*	E1A binding protein p300	1.07	0.618
*FGF1*	Fibroblast growth factor 1	−1.51	0.142
*FGF2*	Fibroblast growth factor 2	−1.50	0.096
*FGF3*	Fibroblast growth factor 3	1.67	0.290
*FGF4*	Fibroblast growth factor 4	2.13	0.217
*FGFR1*	Fibroblast growth factor receptor 1	1.38	0.103
*FOXA2*	Forkhead box A2	−3.43	0.007
*FRAT1*	Frequently rearranged in advanced T-cell lymphomas	1.17	0.317
*FZD1*	Frizzled family receptor 1	2.00	0.127
*GDF2*	Growth differentiation factor 2	2.38	0.235
*GDF3*	Growth differentiation factor 3	1.61	0.382
*GJA1*	Gap junction protein, alpha 1	6.80	0.030
*GJB1*	Gap junction protein, beta 1a	−19.00	0.008
*GJB2*	Gap junction protein, beta 2	1.70	0.214
*HDAC2*	Histone deacetylase 2	−1.12	0.470
*HSPA9*	Heat shock 70kDa protein 9	−1.03	0.991
*IGF1*	Insulin-like growth factor 1	−3.42	0.859
*ISL1*	ISL LIM homeobox 1	−12.77	0.000
*JAG1*	Jagged 1	4.55	0.128
*KAT2A*	K acetyltransferase 2A	2.91	0.003
*KAT7*	K acetyltransferase 7	1.07	0.546
*KAT8*	K acetyltransferase 8	1.92	0.019
*KRT15*	Keratin 15	1.31	0.588
*MME*	Membrane metallo-endopeptidase	−1.05	0.923
*MSX1*	Msh homeobox 1	3.16	0.099
*MYC*	V-myc myelocytomatosis viral oncogene homolog	−1.15	0.941
*MYOD1*	Myogenic differentiation 1	1.83	0.252
*NCAM1*	Neural cell adhesion molecule 1	2.65	0.154
*NEUROG2*	Neurogenin 2	−1.53	0.256
*NOTCH1*	Notch 1	1.91	0.037
*NOTCH2*	Notch 2	1.05	0.626
*NUMB*	Numb homolog	−1.04	0.973
*PARD6A*	Par-6 partitioning defective 6 homolog alpha	2.35	0.027
*PDX1*	Pancreatic and duodenal homeobox 1	2.42	0.225
*PPARD*	Peroxisome proliferator-activated receptor delta	1.37	0.148
*PPARG*	Peroxisome proliferator-activated receptor gamma	−8.28	0.022
*RB1*	Retinoblastoma 1	−1.25	0.113
*S100B*	S100 calcium binding protein B	1.05	0.110
*SIGMAR1*	Sigma non-opioid intracellular receptor 1	−1.09	0.407
*SOX1*	SRY (sex determining region Y)-box 1	1.65	0.304
*SOX2*	SRY (sex determining region Y)-box 2	−1.00	0.046
*T*	T, brachyury homolog	2.61	0.059
*TERT*	Telomerase reverse transcriptase	1.56	0.376
*TUBB3*	Tubulin, beta 3	−1.44	0.183
*WNT1*	Wingless-type MMTV integration site family, member 1	1.53	0.331

*p*-values show the comparison results between the diabetic dermal stem cells (DSCs) and the normal DSCs.
